# Book Reviews

**Published:** 1832-11

**Authors:** 


### CRITICAL ANALYSES.

Quæ laudanda forent, et quæ culpanda, vicissim,
Illa prius, cretâ; mox hæc, carbone, *notamus*.—Persius.

*The Sources of Health and Disease in Communities; or, Elementary Views of "Hygiène," illustrating its Importance to Legislators, Heads of Families, &c.* By Henry Belinaye, Esq., Surgeon Extraordinary to her Royal Highness the Duchess of Kent, &c.—18mo. pp. 261. Treuttel and Würtz and Richter, London.

The truth of the old and well-known adage, that "prevention is better than cure" is no doubt universally admitted; but still, if we look into the legislative enactments of different countries of the present day, or if we refer to the labours of the medical world, we shall be convinced that much less attention is paid in modern times than was in days of yore, either to the mental and bodily training of youth, to ward off diseases from all classes of society, particularly the poor, or to secure, as far and as effectually as human means can secure, a healthy, robust, and consequently happy race of citizens. We may mention one fact as a proof of the comparative negligence which is now shewn for the preservation of the public health in most countries. Russia and Turkey are the only European countries in which there are public baths destined by the government for public use. In our own language, especially, we find but few works on the subject of public or private hygiène. Sir John Sinclair's "Code of Health and Longevity" is, indeed, an elaborate performance, and contains a good abstract of the most interesting facts that had previously been elicited upon these most important subjects, together with much valuable matter from the author's own mint. The essays published by Dr. Beddoes, under the title of "Hygieia," are also very instructive: this writer takes a comprehensive view of the comparative advantages of different systems of education; the diseases which are incidental to different periods of life, and the best means of preventing their occurrence. The work now before us is the only one upon "Hygiène" which has for some years been presented to the profession by an English writer; and although Mr. Belinaye has not entered very deeply into any part of his subject, he has the merit of having added to our bibliotheke a well-written, amusing, and instructive, volume; one, indeed, from which the physician may glean much useful information, the statesman many hints that he would do well to act upon, and the general reader abundant food for conversation upon various topics which are now, in all societies, perhaps from a *choleraphobia*, much more keenly than cunningly discussed.

The purpose of Mr. B.'s essay is to investigate the influence of laws, institutions, habits, climate, &c. on the vigour and health of man; and, with a view to practical utility, to apply the results of these inquiries more particularly, though not exclusively, to this country. The attention of the reader is, first, directed to remote physical influences; those of the heavenly bodies, shewing even their active and continued operation on human existence. Secondly, the singular laws that influence propagation; shewing, amidst some still hypothetical views, how much of positive and available knowledge we have obtained. Thirdly, emanations; both those that arise naturally from the earth, and its productions, and some of those that are elicited artificially. Fourthly, the subject of effluvia is considered; under which head Mr. B. glances at the police of health, and at a great number of sources of diseases, several of which are absorbing topics of disquisition at the present time. Fifthly, a rapid survey is taken of the bearings of civilization on human life; which enables the author to advert to the characteristics of political medicine, and to complete the outline of his first part of Hygiène, which constitutes in itself a separate treatise upon the subjects which it embraces, although more ample illustrations and elucidations may be offered hereafter in a second volume.

Mr. B. truly observes that, from the earliest moment of existence to the last stage of human decrepitude, an influence of the most decided kind may be exerted over mankind; nay, not only from the very threshold of life, even the form and qualities of the future being may be modified by attention to the rules of hygiène. "As changes are unceasingly going on, even in the hardest constituents of the human frame, the constitution of the individual must depend on the media (atmosphere, &c.) in which he is placed; the food from which he is elaborated; the exercise and use made of the nervous energy by the brain, in obedience to the passions of the mind; the health of man, consequently, depending as much on the circumstances acting upon his body and mind, as that of the shrub does upon the sun which vivifies it, and the soil in which it is planted."

In the first chapter Mr. Belinaye considers the subserviency of man to physical influences; and, as the introductory remarks afford a fair specimen of the style in which the work is written, we give them in his own words.

"Previous to the consideration of those branches of our subject which possess a greater number of results immediately applicable, we must prepare for the study by a contemplation of some more general influences. We begin by the first that naturally presents itself, a subject, however, by no means destitute of interest, or of facts capable of useful application.

"In order to feel in its full force the absolute dependency of man on the surrounding elements, we must begin by stripping him of the accessories of his artificial state, of the confidence he derives from his social and collective position, of the glare cast around him by his passions and imagination, and of all the adventitious circumstances by which he is dazzled and misled. We must view him in his naked and comparative insignificance on the surface of the great globe he inhabits; in his mortal frame, no more than a minute and momentary combination of its elements; beautiful, indeed, but quickly resolving itself again into them, in obedience to the common laws that pervade it. As a constituent portion, therefore, of his terrestrial abode, he is not only concerned in its immediate economy; in its productions, of whatever reign, and whether hostile or friendly to his nature; in its forty miles of circumambient atmosphere, in heat, cold, and light, whatever their sources; in the electricity lurking in the clouds above, and in the operations of the internal world beneath him; but he is also a partaker in the mighty and remote influences which govern his planet, and bid it revolve in the fields of space.

"Men have long since agreed to reject the exaggerated opinions entertained by their forefathers, as to the great influence of the heavenly bodies. But as popular opinions can only proceed from, at least supposed, experience, so they are generally found to be grounded on truth, and of practical utility. On the subject of planetary influence, one of the greatest mathematicians of the age has observed, that it would be unphilosophical to doubt the existence of influences, because we cannot explain or test them by any means we at present possess; that we must have recourse to a calculation of probabilities; and that all we have to determine is, how many coincidences of supposed causes with positive effects are requisite to establish our belief.

"Whilst we know that the diurnal changes, the tides, the tropical winds, are affected by sol-lunar influence, the actions of the human body, particularly when diseased, appear likewise to acknowledge, directly or indirectly, allegiance to the same power. We may smile at the blind credulity which induced Tiberius and Varro to submit to the operations of the tonsor only at certain phases of the moon; we may receive with suspicion those observations, which would claim force from analogy, proving the increase and decrease of the size of some animals, the agitation of others, &c., at the time of the full moon; observations supposed to have originated the old custom of shearing sheep, castrating domestic animals, &c. only at certain of her phases. Even though we may consider with some distrust the power of lunar influence asserted by some on the duration of gestation and incubation, and on the recurrence of the peculiar function of women, still we have a multitude of facts that induce us to believe in that power, either alone or in combination with the sun, on human life. Chancellor Bacon was subject to syncope during eclipses of the moon; Matthew Faber speaks of a gentleman who always spent the days preceding an eclipse buried in thought and melancholy, and who, when it took place, rushed headlong into the streets, striking with his sword every thing he met. A young girl is reported by Hoffmann as labouring under an extraordinary painful swelling, beginning and finishing with the increase and decrease of the moon, &c.

"In all parts of the globe, men have thought they recognised lunar influences on the crises and exacerbations of disease. The phenomena of invasion and duration of fevers appears to be controlled by that influence. This is seen more distinctly as we approach tropical climates, where generally the operations of nature partake of a development very favorable to scientific investigation. Dr. Jackson states, that in Jamaica the febrile intermittent and lunar periods correspond. Dr. Lind observed the same phenomenon, and adds, that deaths occur mostly during the ebb of the tide; and that eclipses produce dangerous relapses in those ill of fever. Dr. Balfour, in the Asiatic Researches, says, that when the sun, during the equinoxes, by approaching the equator, increases, by his power, the attraction of the moon, with the increase of the tides occur also the increase of violent fevers and mortality.

"The sun, being the centre of our planetary system, has also independent and more extensive influences. Without recurring to the more minute or apocryphal, we will advert at once to those exerting powerful effects on the human frame; although the reader, curious in such matters, may find in scientific publications many very remarkable examples of the former, persons losing daily all powers of vision at sunset; and, amongst others, that mentioned by M. de Humboldt, of a noble lady, the Countess de K——r, who lost her voice when the sun disappeared, and only recovered it at his rising in the morning.

"By the variation of the relative position of the sun in the ecliptic are produced the seasons; and by the seasons is the human frame greatly influenced. Statistical reports prove that the incipient life of the human being, as well as its death, are subject to their power.

"Conception, in women, does not take place with equal facility at all periods of the year; the difference is marked, although it may be modified by climate. The same laws are only more inflexible in animals, because the circumstances of passion and artificial temperature are not, as with men, at their own command. However, the lower classes of society, whose propensities and whose circumstances bring them nearer to a state of nature, are more decidedly governed by the law of preference as to seasons. The end of the spring, and the beginning of the summer, are the periods that in our climates tend, in a remarkable degree, to the increase of population. Taking for our guide the more correct reports of France, the most prolific months, drawn from accurate registers, may be arranged, according to the order of the relative number of births in each, thus: March, January, February, May, August, October, September, July, November, June, and December. Since, then, the greatest number of births occur in March, January, and February, it must be obvious that the months most favorable to conception are June, April, May, &c.

"The tables of mortality in England have also shewn that the preponderance in the number of deaths is regulated by the season, and that those months commonly considered the most fatal are not so in fact. We sometimes overlook the circumstance that the mortality of any given season is often only the result of the character of that which immediately preceded it. If, as it frequently happens, an unusually cold winter succeeds to a very hot autumn, the same effects are produced as if we had suddenly removed from a hot to a cold climate. Reflections of this nature suggest salutary precautions.

"The heat and light of the sun can scarcely be correctly separated; however, we will consider the former under the chapter of Climate. A few words only can be devoted to the subject of Light; and they are offered because people generally think so little of those phenomena around them, to whose influence they are habitually exposed. In the same manner that persons who are continually singing and talking are the least aware of the importance of those exercises to the human economy, improving the health of those who have been condemned to silence and solitude, or killing those who suffer from diseases that require rest; so those who enjoy the purest rays of light are those often the most blind to its powerful effects.

"It is not merely that the eye, anxiously accommodating its powers to its privations, and enabling the prisoner to distinguish objects in what appears to us the densest darkness of his cell, will be struck blind by the splendor of day when he comes forth, as many others are by the too vivid brightness of lightning; but the light of the sun appears, as it were, to feed the human body. Removed from it, the body grows pale, exhausted, and bloated; scrofula, and many other complaints, depending upon a want of tone, are generated. In this respect we are not unlike plants, which we see, when forgotten in a cellar, grow blanched and sickly; shooting out rapidly their feeble and flexile stems, nor resuming any thing of their distinctive colour until some leaf can expand itself eagerly towards the light of a crevice.

"Among human beings it is not, unhappily, those only who are condemned for their crimes to seclusion who prove to us the injurious effects of the absence of light. The unfortunate beings doomed to work for us in the bowels of the earth are living illustrations of this fact; but those who inhabit large cities have still nearer evidences: in the dark and narrow streets and lanes, in the windowless houses, in dark kitchens and cellars, too many, particularly of the infantine and young, confess the absence of that influence that imparts the beautiful hues to the human cheek, as to the varied creations of nature, and which, combined with heat, sets a distinctive mark on the natives of different parts of the globe. Considerations of this kind might lead legislators to pause before they exclude, by taxation, any portion of a blessing, which is indeed one of the vital principles of animated existence.

"That which might best compensate the absence of this stimulant is that which those so deprived are least likely to obtain, abundant and nutritious food." (P. 17.)

A few brief remarks follow on the influence of electricity on the human frame, and next the author refers to the effects of different modifications of sound. "Sound is to the ear what light is to the eye: they both contribute beneficially to stimulate the human system, besides their important agency in the functions of relation." As we find domestic animals, reptiles, and spiders, so acutely alive to the power of music, and have, in the Philosophical Transactions, a record of notes which greatly agitate even wild beasts, we are prepared to understand some of its effects on man.* Various curious facts are referred to in the work, in proof of the powerful influence produced upon the mind and nerves by sonorous percussion.

Chapter II. and III. *Laws of Propagation.* In these chapters Mr. Belinaye proceeds to make good his previous assertion, that there are natural laws which enable us to influence and modify the propagation of the human species; and the opinion is supported by many curious facts and ingenious arguments.

Chapters IV. and V. *Emanations.* The consideration of the subject of the atmosphere, in its many aspects, and in its important relations to the life and welfare of man, is deferred to another place; but here is premised the office it performs in regard to the emanations constantly arising around us.

"In this respect, Fodèré has justly observed that it resembles water, which immediately takes up, and becomes the vehicle of, all soluble matters that it can meet with.

"At the same time, how large a portion of the materials of our sphere may the atmosphere act upon in this manner; since the fluid, the gaseous, the solid, forms, are only comparative states of bodies, depending upon the degree of heat to which they are submitted at any given time, or in any given situation, and which varies constantly. Even what is habitually fluid in our planet, may be habitually solid in one further removed from the great centre of our system, and vice versâ.

"'The atmosphere of spots inhabited by living beings is, if we may be allowed the expression, but a confused assemblage of all that has passed from the solid to the fluid, or to the soluble state, through the agency of heat; of effluvia exhaled incessantly from the bodies which the air surrounds, penetrates, dissolves; odoriferous matter, consisting of numberless molecules emanated from perfumed bodies; water vapourised, or in a state of suspension; elastic fluids, constantly produced by new combinations; smoke, arising from the burning of so many different combustible bodies; of dust, thrown into the bosom of the atmosphere by so many arts of necessity, and by friction; all of which, carried away to some distant spots, are destined to become the nuclei of new bodies.' (*Fodèré*, vol. vi. p. 285.)

* We may here refer our readers to a chapter in that very interesting miscellany, the "Curiosities of Literature," by D'Israeli, entitled "Medical Music."—Editors.

"But it is when the weather is warm and the air charged with moisture, that the atmosphere performs most readily the office of taking up and disseminating odours and effluvia; a circumstance which renders the study of this subject peculiarly important in England, where dampness is so common, and sudden transitions of temperature frequent, even in the greatest depth of winter. Few there are so unblest as not occasionally to have experienced the delightful fragrancy of a garden, when the warm air has become moistened by the dews of evening or of a fecundating shower. But fewer still of such as visit the haunts of poverty have escaped the sickly odours that rise upon the damp warm air of the closer districts of the town.

"Before speaking of the odours and effluvia of which the atmosphere is the ever-ready vehicle, we must not forget to observe the effect of those sudden molecules or particles elicited and thrown constantly into the air by friction of artificial processes of manufactories, &c. Dust, or minutely divided substances, produce the most sensible and pernicious consequences, when inhaled constantly or in large quantities. The eyes, the mucous membrane of the air-passages, and the skin, suffer severely from dust, whether it be merely from the mechanical irritation or from the peculiar pungency of the pulverized substance. Hence the workmen belonging to the numerous trades, in the operations of which a degree of dust is unavoidable, are so often affected with coughs, consumption, asthma, hœmoptysis, &c. In the large towns, a great improvement has been introduced in the streets, that which is called macadamization; but if care be not taken to remove, during wet weather, the loose mud of the surface, before dry heat and the friction of carriages turn it to powder; if, during dry weather, the surface be not regularly watered, and that sufficiently to keep down the dust, during the whole of the day; if these precautions, we repeat, be not taken, the fearful annual average of deaths from diseases of the lungs in the bills of mortality will be inevitably increased by the irritation which the powdered granite, borne in the atmosphere, must necessarily engender in the respiratory organs.

"Although not strictly within the limits of our present subject, we shall take this opportunity of observing the danger incurred by delicate persons going out in the evening of a hot day, when large macadamized streets are watered: the cold and dampness of the atmosphere, produced by the evaporation, may prove very prejudicial.

"The evidence afforded by needle-makers and stone-grinders would lead us to believe that dust reaches to the extremest ramifications of the bronchiæ. We may also mention here, 'en passant,' that it is not advisable for the consumptive to travel along dusty roads, and particularly in dry hot winds: this they are often made to do in search of health, amid foreign climes. At such moments they should rather reside amidst green fields at home, or only travel, if possible, by sea, (a mode of conveyance notoriously and peculiarly salutary,) or in weather when the roads are free from dust." (P. 89.)

The fifth chapter contains some interesting remarks on the subject of *odour*, the object of which is to shew the influence produced by this kind of emanation over the vital functions, and to convey an idea of the importance of exhalations in a more extended view.

The four succeeding chapters treat on the very important subject of *Effluvia.* To arrive at just conclusions, says Mr. B., on the subject of human effluvia, we must not forget the office which vitality performs in the living being.

"By vitality, we mean that mysterious principle which baffles all research, and must ever involve the art of medicine in some uncertainty; which quickens, developes, and circumscribes all the various forms of animate nature; which preserves each of them in its individual integrity, against the encroachments of the rest; the same which endows an acorn with the primordial of the strength and majesty, and of the hundred years of an oak, which, in a germ apparently of the same magnitude, only enfolds the ephemeral beauty of a flower; that, finally, which assigns his degree of perfection and his term of endurance to man. It is on this particular principle, which we must be aware is more or less powerful in different individuals, that not only the vigor, but also the purity, of the body must depend.

"Let us consider, for one instant, the effect of its presence or absence. Whilst the blood is in the veins of the arm, its globules move on in an unceasing stream, it is homogeneous and fluid; the lancet is plunged into the arm, and the blood runs into a cup: its constituents separate rapidly; the charm of vitality, which held them together, is dissolved. Here we have water, there a coagulated cake, and in another place the colouring matter. The whole mass ultimately, and not slowly, putrefies. It is, then, vitality which preserves the animal frame from this abject and complete submission to external agents, nay, even from internal ones too; for it is found that, if a rabbit be killed instantaneously, the gastric juice, destined to digest its food, will digest the tissues of its own stomach.

"But it is only when life is completely withdrawn that the human body tends to putrefy. Vitality, like another principle equally enigmatical, the mind, is liable to become either suddenly or gradually impaired: with this change the septic tendency begins. Bad or little food, fatigue, want of air, light, or of any other element upon which vitality depends for its support, will, by diminishing its conservative power, abandon the body to the inroads of putrescency. Hence, from the moment it begins to droop, its effluvia become more and more acrimonious, until death deliver it up entirely to the ravages of putrescency. It is when vitality is affected that the body throws out the most noxious effluvia, and it is particularly open to receive the germ of every foreign contagion. Thus, the virulence of one cause conspiring with that of another, it spreads through all classes of the community the disastrous and awful consequences of morbid influences. Such of the lower classes as are most subject to depression of vital power afford a permanent hot bed to the habitual contagions of each country, and an inviting resort to others, which either arise spontaneously or are conveyed from distant lands by the intercourse of man, and add so awfully to the sum of disease and mortality.

"Could, then, the vigor of the lowest classes of society be maintained, not by luxury, but by an equable supply of the necessaries of life, earned by regular labour proportioned to their strength, epidemics and contagions would either sweep by innocuous or prove comparatively harmless. This must be an additional incentive to the paternal care of governments, and to the charity of the affluent classes. Without this, legal enactments, whether for exterior or interior protection, must prove comparatively nugatory, dealing, as they do, with a power that travels on the wind, or is generated with most irresistible virulence in the obscurest corner of a dungeon, or in the lowest abode of human misery. Without this in vain will the affluent classes fence themselves round, and lock themselves up from the ordinary intercourse of the community; they will but have increased the sum of misery, and at the same time the momentum by which the disease will be enabled to reach them. Health and disease are in immediate connexion, as we have proved elsewhere, with good or bad government, general prosperity, or partial affluence. Nor should we forget that selfishness affords no escape. Liberal ideas on these subjects are the result of civilization, and, generally speaking, the civilization of the globe may be measured by its tables of mortality.

"The very protection afforded to us in nature by the principle of life, is at once a proof of its existence and an illustration of its character. When putrefaction has reached its most pernicious stage in any substance, the generation of myriads of animated beings commences, some visible to the naked eye, and still more only to be detected by the aid of the microscope. So common, indeed, is the generation of living creatures in putrefactive matter, that the ancients considered putrefaction as one of the causes of life. Thus, by the rapidity and extent to which animalcular reproduction is favored by putrescency, nature endeavours to preserve us from its deleterious effects. In spots in the hot tropical climates, where, as soon as a certain degree of humidity prevails, the most deadly effluvia arise, on the fall of a shower the rich mould appears vivified." (P. 115.)

Mr. B. next refers to the discussions and contentions which arise on all sides, as soon as some new disease is imported from abroad, or arises in some spot at home, as to its having simply an epidemical character or one that is contagious, or both. We agree entirely with him in the belief that the contagious, as well as the malignant, character of diseases, depends mostly, if not entirely, upon the degree of vital energy, and the narrowness of the space,

&c. within which those who suffer from it are confined. "In the narrow streets, in the dark blind alleys, and small rooms, where human beings are found, of immoral and filthy habits, ground down, moreover, by poverty, labour, and misfortune, by every thing, in a word, that affects vitality; in such places it is that epidemics first appear, and then grow into contagion. If persons who can command comforts and conveniences are attacked by the invading disease, its contagious character disappears, or no longer betrays itself; and then it is rashly pronounced to be *only* an epidemy, or disease from local miasmata or influences." In our judgment, these opinions are as correct as they are impartially stated. It is true they may not please either of the two parties into which, with some few exceptions, the medical world appears recently to have been divided, the exclusive contagionists, or the equally exclusive non-contagionists; but, notwithstanding the host of opponents Mr. Belinaye's neutrality may raise up against him, we feel convinced that he takes a rational and right view of the question.

The eighth chapter is dedicated to the subject of the virulence of effluvia from the bodies of the dead. Mr. Belinaye adverts to the many instances on the continent of persons sacrificed by a premature opinion of their being dead. Buchier states the following facts: "Out of 180 examples of persons erroneously supposed to be dead, fifty-two had been buried alive; four had been opened after supposed death; fifty-two had spontaneously revived after being put into their coffins; seventy-two were discovered to be alive after having been deemed dead." Even in the time of Pliny alarm had began to be felt on this subject, and he dedicates a whole chapter to it. Surgeons have, through inadvertence, opened bodies, which have only parted with life on the application of the scalpel: this occurred to Vesalius. In 1763, a clergyman, supposed to have died from apoplexy, groaned when wounded by the knife of the anatomist. La Place was informed of the circumstance, and asked what was to be done? he replied, "Gemir et se taire."

"Honourable to the feelings of the nation, as all must consider the procrastination of interment in England, it is not without its bad consequences. The effluvium of a dead body, diffusing itself in a house, where the minds and vital energy of its occupants are depressed by sorrow, and where the distressed relatives, perhaps, refuse necessary nutriment, may produce the worst effects. To parry these evils, and the still more awful errors of interring the dead alive, a consultation of competent persons might be appointed to examine the dead, as soon as possible after decease, and decide on the measures to be adopted. The civil law of France has made an enactment on this subject, which, if strictly adhered to, would go far to prevent these three evils, crime, burying the living by mistake, and keeping the dead to infect the living.

"So deeply and awfully have some people been impressed with the horrors of premature interment, that, in one of the old imperial towns of Germany, a plan has been devised and adopted, as a security against this, as well as the other evils we have enumerated. Every person, after death, is carried to a well-ventilated room, constructed for that purpose, near the church; the corpse is warmly covered, and laid upon a table, the hands connected with strings, communicating with bells suspended in an adjacent room, where a watchman is constantly on duty. To ensure his vigilance, he is compelled, every quarter of an hour, to advance the finger of a dial, which will only move at that interval of time. We relate this from recollection, which, however, is accurate in all essential particulars. Two persons were saved by this expedient." (P. 165.)

Mr. Belinaye mentions many facts in proof of the injurious effects which arise from the effluvia of dead bodies; and he very properly condemns the customs of England with respect [illegible] burying the dead either in cemeteries in populous places or in churches.

The ninth chapter, in which the subject of Effluvia is continued, contains many excellent suggestions as to the best means of diminishing the destructive effects of epidemic diseases, and of securing the salubrity of large and thickly populated cities.

Chapter x. *Civilization.* In many respects, a refined state of society militates against the health of a community. "The substitution of art for nature, the excitation of the passions, the overstraining of the mental powers, the many arduous professions, and, still more, the many new trades, are among the causes that, in civilised states, produce in the human being fatuity or madness, render him feeble or deformed, and still more frequently curtail his existence." In what manner these evils occur, and how they may best be palliated or removed, Mr. B. hopes at some future period to indicate. At present the attention of the reader is solicited to a different and more agreeable prospect, to the influence of civilization, notwithstanding the evils just enumerated, in contributing to the aggregate health and years of man.

"One of its prominent merits, the happy result of the reasoning powers acting upon the accumulated observation of man, is Hygiène, whose intimate connexion with civilized life, and influence over it, must not be overlooked. In the lowest state of society, indeed, men must have some glimmering of its value, and may seek to discover its secret springs of health; but it is in the bosom of civilization alone that it can be made to pour a beneficent stream, augmented by the general contributions of science and of art. * *

"We see that civilization, developing the powers of mind, cannot be inimical to health. But it is more important for our practical purposes to consider other arguments in its favor, and thus, taking a country in the state in which it may first be discovered, by navigators, what do we find? In the warmest and most fertile regions, immense forests, jungles, or marshes, cover the land; the former impervious, from the entangled growth of underwood, or dangerous from their reptile inhabitants; the jungles, inhabited by tigers; and the marshes fermenting with animal and vegetable remains. Death there triumphs; and if man be found amid such scenes, scarcely superior to the beasts with whom he disputes his food, and possessing but a few rude and insufficient weapons to attack his prey, he almost starves amid the wild luxuriance that surrounds him.

"But the scene changes. The navigator has announced his discovery; has described the wealth of the untouched soil, the resources of its climate, the unclaimed riches of its interior; and colonists have ventured to the land of promise. The forests now are felled and burnt; the sun and air admitted; the land is cleared. The reptiles, and its other noxious possessors, disappear; the marshes are drained; and the soil, exposed to the solar heat and purifying winds, becomes, instead of the destroyer, the fruitful supporter of human life, and the bounteous rewarder of human industry.

"But the arts of civilization stop not here. The domestication of animals, and the cultivation of individuals of the vegetable kingdom, alter, as well as improve, to such a degree, their appearance, that, in the course of time, the eye of the learned botanist or zoologist alone can detect their original types. The size and colour of the animal are changed, and its flesh rendered tender and digestible, by domestic management, by crossing of breeds, by peculiar nurture and food, by spaeing the female, and castrating the male, &c. By means no less familiar to the cultivator, the sloe becomes the luscious plum, the stunted and acrid crab grows into the glowing apple, and plants of a poisonous class, as the potato, become the main nutriment of nations.

"But if the most luxuriant nature be an useless gift to man, without the arts of civilized life, it is equally certain that a country which, in the palmy state of social superiority, supported a dense population at home, and exchanged its superabundance for foreign luxuries, may become, by degradation from that 'high estate,' incapable of furnishing the same population with the bare means of existence. Many are the fertile lands disappearing beneath the incrustation of new substances, allowed constantly to form and accumulate. On beholding vast steppes or desarts, or seas of moving sands, how difficult, at first, is the belief that, in wilds so desolate and barren, nations formerly existed, whose glories still illumine the page of history!

"Egypt, whose wondrous structures, whose stupendous pyramids, whose population and commerce, whose arts and sciences, in a word, whose civilization, in its fullest sense, have been the admiration and wonder of all succeeding ages, the land of Egypt is fast disappearing beneath the invasion of sand. Every succeeding year adds a new stratum; and the sand now, in some places, blows fairly into the Nile itself, the source whence all the fertility of the land is derived. Had this destructive tribute been buried yearly as it came, by the plough or the spade, mixed with manure, and still covered by its periodical irrigation, it might, even in our times, have maintained a nation, enlightening the world in peace, and subduing it in war." (P. 212.)

What has been observed of remote countries but recently discovered, and of nations now living but in history, is everywhere equally applicable. In the settlements of North America, disease and mortality are receding before the blows of the axe, and the burning of the trees it fells. Wolves, which in 1740 still existed in Ireland, are now altogether destroyed in the British islands; other noxious animals, which prey upon the resources of man, are disappearing from Europe; and in some of its most marshy lands, those of which the thrifty Dutchman has defrauded the sea, and which he only retains by his dikes and by pumps worked by windmills, the effects of constant cultivation have powerfully counteracted that malaria which at Walcheren, some years back, nearly destroyed an English army.

"The reverse of this picture is presented by some of the finest cities in the world. The neglected Pontine marshes, where the life of the miserable inhabitants may more properly be styled a lingering death, once supported a people vigorous in mind and body, and imperishable in history. From hence has Fate advanced to destroy the cherished pride of many ages. Rome, once the queen of cities, but foredoomed to the fate of Babylon, is daily diminishing in population. The malaria advances from street to street, and has already become the sole tenant of some of its finest temples, churches, and palaces. Rome, indeed, might be singled out as offering in itself a history of most that is interesting in the police of health. When still the capital of the world, in spite of the malaria, she overflowed with population; and her disadvantages were counteracted by the activity and moral excitement of her inhabitants, the drainage of marshes, the width of streets, the abundant supply of pure water from forty aqueducts, &c." (P. 218.)

Mr. Balinaye introduces many interesting facts, for the purpose of shewing how much the abundance and nature of the food of man, and the difference in the productions of the soil, influence his physical powers, development, and conformation.

"The difference in the productions of the soil which form the principal food of man in different provinces, is one of the reasons why, in the recruiting of armies, the quota of men supplied by each district is found to vary so much in proportion from each other. In some territories fewer births take place in proportion to their extent; in others, more female than male, or vice versâ. The physical development or conformation also probably varies according to the soil. Charts have been made in some countries, to shew in what districts recruiting parties can be placed with most advantage, where men are found of a sufficient height for grenadiers and cuirassiers; where they are adapted for light cavalry and infantry, &c.

"These are facts of the same universal character as those formerly mentioned, which go to prove that longevity conduces to civilization and to the superiority of nations; that monogamy, by maintaining the proportion between the sexes, of twenty-one to twenty-two, contributes no less to effect these desirable objects of governments, &c.

"Observations of this nature lead us naturally to seek, and reflect on those recondite, general, fixed principles by which the welfare of mankind is governed; principles which, while they hold a sway over human existence, the circumstances in which they obtain are still susceptible of certain modifications by human will." (P. 229.)

The value of civilized resources, and the importance of hygiène, are shewn by the great change in the rate of mortality in the British naval service. Formerly, twenty or thirty persons a day were not uncommonly destroyed by scurvy on board ship; but now, by the administration of citric acid, of the juices of plants, accompanied by many observances of hygiène, the British navy enjoys a greater freedom from disease than any other class in the community.

We must close our notice of this very interesting little volume with one more extract.

"The vigilance of a government, enlightened by the scientific bodies of the state, and seconded by the influence and exemplary habits of the better classes, and the industry of the rest, can do more for the healthiness of a country than all its natural advantages, (as we shall presently shew from statistical facts,) and all the science of the most gifted medical men, dispersed in ever so great numbers throughout the community. Most of the elements with which a government can act were, till lately, wanting. England changed from a despotical to a free state at a period when science was too little advanced for the nation to have felt the advantage of keeping in the hands of government the direction of all that relates to the health of the people. It is but lately that a correct census has been published, with tables of mortality and statistical reports on the health of the nation, that could afford a basis of reasoning. The mere possession of such materials suggests conclusions of the most important bearing on the welfare of all, and has opened the eyes of influential men to the conviction how well grounded was the esteem in which hygiène was held by continental nations." (P. 242.)

Mr. Belinaye informs the readers, in his preface, that it is his intention to pursue his subject in a second volume. By that which we have just analysed, he has very favorably introduced himself to the public and the profession, and we cannot doubt that he will meet with ample encouragement to induce him again to take up his pen.

*Organographie Végétale; ou, Description raisonnée des Organes des Plantes, pour servir de suite et de Développement à la Théorie élémentaire de la Botanique, et d'Introduction à la Physiologie Végétale, et à la Description des Familles; avec* 60 *Planches en taille douce.* Par M. Aug. Pyr. De Candolle, Membre du Conseil souverein de la République et Canton de Genève, &c. &c. &c. 2 vols. —8vo. pp. 558 and 304. Paris, chez Deterville.

*Conversations on Vegetable Physiology; comprehending the Elements of Botany, with their Application to Agriculture.* By the author of "Conversations on Chemistry," &c.—2 vols. 8vo. pp. 288 and 304. Longman and Co., London.

*Physiologie Végétale; ou, Exposition des Forces et des Fonctions Vitales des Végétaux, pour servir de Suite à l'Organographie Végétale et d'Introduction à la Botanique geographique et agricole.* Par M. Aug. Pyr. De Candolle.— 3 vols. 8vo. pp. 1580.

*An Introduction to Botany.* By John Lindley, F.R.S. L.S. G.S., Member of the Imperial Academy Naturæ Curiosorum, of the Botanical Society of Ratisbon, &c. &c. &c. *With six Copperplates and numerous Wood-Engravings.*—8vo. pp. 557. Longman and Co., London.

Since botanists, escaped from the thraldom of merely artificial systems of study, have estimated the (in one point of view) admirable Linnæan analytic scheme more according to its absolute and intrinsic merits than they formerly were wont to do, and have ceased to regard this instrument for the acquisition of knowledge, which its immortal author placed in their hands, as the science which by its means may be acquired, a very marked improvement has been observable in the botanical works which have issued from the press; for we no longer find authors of celebrity asserting that with the *culture and uses of plants* "a botanist, as a botanist, has nothing to do;" but, on the contrary, the "amabilis scientia" has been allowed to resume her fair proportions, of which she had long been most miserably curtailed, and the most able botanists are the most industrious labourers, and the most actively employed in rendering the truths revealed by science beneficial to man, and applicable to the common purposes of life: and the advantages which both horticulture and agriculture have derived from the scientific direction of their practice, founded on the principles of botanical philosophy, our fields, our gardens, our markets, and our tables, afford convincing proof. Botany is no longer a science of hard names, and the attention of botanists is no longer chiefly confined to the distinction and arrangement of plants; for, without neglecting the systematic branch, the physical department is ardently studied, and the extraordinary facts which the study of vegetable anatomy and physiology discloses, well reward all those who devote themselves to these experimental investigations.

Among the many who have wrought faithfully and successfully in the service of science, the authors whose names now head our pages hold no middle rank, and their present works deserve no measured praise. We have long reproached ourselves for not having hitherto reviewed the first, and of the second and third we were preparing a notice when the fourth arrived most opportunely for our purposes.

The five volumes with which we have been favored by De Candolle contain a complete exposition of modern botanical philosophy, as far as the structure and development of the organs of plants, and the functions of these organs, are concerned. They are, however extensive as they seem, but the two first parts of that learned botanist's "Cours de Botanique," and the heralds of the geographical, agricultural, and other sections; hence, important and invaluable as they undoubtedly are, the scale is too extensive for the early student, and too recondite for the world at large. A more popular and a more scholastic work, a condensation of the contents of De Candolle's volumes, with reference to the subjects on which he has not yet published, was much wanted; and this is now in part supplied by Mrs. Marcet and Mr. Lindley, both of whose works are in great measure founded on those of De Candolle: the first, indeed, being confessedly derived from notes taken during attendance on a course of the Professor's lectures; the second, however, though following the general principles of De Candolle's scheme of investigation, and drawing largely on his volumes, contains much matter collected from other sources, and much likewise that is original.

We have, therefore, looked through this "Introduction to Botany" with pleasure, and, although not agreeing with its learned author in every point, we can safely recommend it as a very able digest of modern discoveries, and likely to be found a very useful manual for students.

This volume is divided into seven books, or sections, respectively devoted to, 1, Organography, or the structure of plants; 2, Physiology, or plants in a state of action; 3, Taxonomy, or the principles of classification; 4, Glossology, or the adjective terms used in botany; 5, Phytography, or the rules to be observed in describing and naming plants; 6, Geography, or the distribution of plants upon the surface of the globe; and, 7, Morphology, or the metamorphosis of organs.

When, in so comparatively small a space, so many and such extensive topics are discussed, it is evident they must be so succinctly treated, that further abridgment would be scarcely possible, and that extracts would give but a very imperfect notion of the contents. They could merely afford a general notion of the manner in which the various subjects are treated, and for such purpose only will the following be given. We would fain have quoted the passages on the different structures of Exogenous Stems, but they could be scarcely intelligible without the figures. They are peculiarly interesting, as shewing that the normal development in certain trees is very different on different sides of the original pith: we have a specimen which is still more decided on this point than any our author has figured; it is a portion of an unknown climber, given to us by our friend Dr. HANCOCK, in which the annual depositions are not only most on one side, but altogether unilateral. And from this, as well as other nearly parallel observations, we had been led to a similar conclusion to that so well expressed in the following quotation as to the age of the Baobabs, and some other trees, which we have always contended have been calculated upon very insufficient and unsatisfactory data; and we recollect very much offending an enthusiastic professor of geology by our public expression of this belief.

"Each zone of the vascular system of an Exogenous stem being the result of a single year's growth, it should follow that, to count the zones apparent in a transverse section, is sufficient to determine the age of the individual under examination; and, further, that, as there is not much difference in the average depth of the zones in very old trees, a certain rate of growth being ascertained to be peculiar to particular species, the examination of a mere fragment of a tree, the diameter of which is known, should suffice to enable the botanist to judge, with considerable accuracy, of the age of the individual to which it belonged. It is true, indeed, that the zones become less and less deep as a tree advances in age; that in cold seasons, or after transplantation, or in consequence of any causes that may have impeded its growth, the formation of wood is so imperfect as scarcely to form a perceptible zone: yet the learned De Candolle has endeavoured to shew, in a very able paper, 'sur la Longévité des Arbres,' that the general accuracy of calculations is not much affected by such accidents; occasional interruptions to growth being scarcely appreciable in the average of many years. This is possibly true in European trees, and in those of other cold or temperate regions, in which the seasons are distinctly marked: in such, the zones are not only separated with tolerable distinctness, but do not vary much in annual dimensions. But in many hot countries the difference between the growing season and that of rest, if any occur, is so small, that the zones are as it were confounded, and the observer finds himself incapable of distinguishing with exactness the formation of one year from that of another. In the wood of Guaiacum, Phlomis fruticosa, Metrosideros polymorpha, and many other Myrtaceæ, for instance, the zones are extremely indistinct; in some Bauhinias they are formed with great irregularity; and in Holböllia latifolia, some kinds of Ficus, certain species of Aristolochia, as A. labiosa, and many other plants, they are so confounded that there is not the slightest trace of annual separation.

"With regard to judging of the age of a tree by the inspection of a fragment, the diameter of the stem being known, a little reflection will shew that this is to be done with great caution, and that it is liable to excessive error. If, indeed, the zones upon both sides of a tree were always of the same, or nearly of the same, thickness, much error would, perhaps, not attend such an investigation; but it happens that, from various causes, there is often a great difference between the growth of the two sides, and, consequently, that a fragment taken from either side must necessarily lead to the falsest inferences. For example, I have now before me four specimens of wood, taken almost at hazard from among a fine collection, for which I am indebted to the munificence of the East India Company. The measurements of either side, and their age, as indicated by the number of zones they comprehend, are as follows:

| | Diameter of Side A. | Diameter of Side B. | Total. | Real Age, or No. of Zones. |
|---|---|---|---|---|
| Cornus capitata ......... | 9 lines. | 36 lines. | 45 lines. | 40 |
| Pyrus foliolosa ......... | 8 lines. | 22 lines. | 30 lines. | 36 |
| Magnolia insignis ...... | 11 lines. | 20 lines. | 31 lines. | 17 |
| Alnus nepalensis ...... | 11 lines. | 23 lines. | 34 lines. | 8 |

Now, in the first of these cases, suppose that a portion of the side A were examined, and the observer were told that the diameter of the whole stem was 45 lines, he would find that each zone was 0.45 of a line deep, and that, consequently, the tree had been twenty years forming a radius of 9 lines, or increasing 18 lines in diameter: the total diameter being 45 lines, he would necessarily infer that the age of the tree was one hundred years; its real age being only forty, as indicated by its zones. And so of the rest.

"When we hear of the Baobab trees of Senegal being 5150 years old, as computed by Adanson, and the Taxodium distichum still more aged, according to the calculations of the ingenious M. Alphonse De Candolle, it is impossible to avoid suspecting that some such error as that just explained has vitiated their conclusions." (P. 64.)

We extract from De Candolle's "Physiologie" a tabular view of the calculations referred to.

"Baobab trees of the following ages have the following diameters and heights:

| Year. | Diameter. | Height. |
|---|---|---|
| 1 | 1-1½ inch | 5 feet |
| 20 | 1 foot | 15 — |
| 30 | 2 feet | 22 — |
| 100 | 4 — | 29 — |
| 1000 | 14 — | 58 — |
| 2400 | 18 — | 64 — |
| 5150 | 30 — | 73 — |

Baobabs have been measured exceeding 90 feet in circumference, that is, larger than the largest in the tabular computation; and which hence must be, if such calculations are correct (!), nearly, if not quite, 6000 (?) years of age: and, if such be the case, how old must the Montezuma cypress be? (Cupressus disticha, Linnæus; Taxodium of Richard, and Schubertia of Mirbel,) which measures 117 feet 10 inches (French) in circumference; i.e. 37½ feet in diameter, and is about 100 feet in height? It must be so old that it would be unpardonable to ask any further questions.

---

*Theory of the Inverse Ratio which subsists between the Respiration and Irritability, in the Animal Kingdom.* By MARSHALL HALL, M.D., F.R.S.L. and E. &c. &c. (*From the Philosophical Transactions.*)

WE have read Dr. HALL's paper with much interest and attention, and trust he will continue inquiries so physiologically important and so auspiciously commenced. Much as we seem to know, and much as we really do know, of the phenomena of life, much we know imperfectly, and very much more remains utterly unknown. Hence there is a very wide field open to philosophical investigation, and we know not any one better calculated to cultivate it advantageously than the author of the present memoir, who thus, as it were, brings us the first-fruits of his research.

So new are the lights which are thrown by Dr. Hall's experiments on the doctrine of irritability, that we shall make no apology for quoting largely from his memoir.

"The object of the investigation, of which the present paper details the principles, is to trace a peculiar law of the animal economy, through the various series, forms, and conditions of animated being. This law may be announced in the following terms:

"*The quantity of the Respiration is inversely as the degree of the Irritability of the muscular fibre.*

"It will be necessary, in the very first place, to define the terms which I am about to employ. The expression inverse ratio is not used in its strict mathematical sense, but merely to designate the general fact, that, in cases in which the quantity of respiration is great, the degree of irritability is low; and that in cases in which the quantity of respiration is small, the degree of irritability is high. By the quantity of respiration, I mean the quantity of oxygen gas consumed, or exchanged for carbonic acid, in a given time, by the animal confined in atmospheric air." (P. 1.)

Dr. Hall very properly observes, that "the word *irritability* only expresses one half of the property or function of the muscular fibre, its susceptibility to the influence of irritants or stimuli; the term *contractility* is equally defective, expressing only the other half of that function, viz. the effect of that susceptibility under the actual influence of stimuli. The designation *irrito contractility*, he adds, would express the whole phenomena. The preliminaries being settled, he continues,

"Organic life appears to result from the impression of stimuli upon parts endued with irritability. The principal stimuli in nature are air, food, and heat; the principal and corresponding organs of irritability are the heart, the stomach, and the muscular system in general.

"The animal series consists of beings variously modified by the varied degree of irritability, and by the varied quantity of stimulus. Throughout the whole these observe an inverse ratio. The bird tribes and the mammalia are characterized by great respiration, whilst the irritability of the muscular fibre is low; the reptiles, the batrachia, and the fish tribes, on the other hand, are endued with a high degree of irritability, and little respiration. The higher parts of the zoological series consist of animals chiefly characterized by the appropriation of a great quantity of stimulus; the lower, by the high degree of irritability of the muscular fibre. The former are animals of stimulus, of activity; the latter are animals of irritability.

"The due actions of life, in any part of the zoological series, appear to depend upon the due ratio between the quantity of atmospheric change induced by the respiration, and the degree of irritability of the heart: if either be unduly augmented, a destructive state of the functions is induced; if either be unduly diminished, the vital functions languish and eventually cease. If the bird possessed the degree of irritability of the reptile tribes, or the latter the quantity of respiration of the former, the animal frame would soon wear out. If, on the contrary, the bird were reduced to the quantity of respiration appropriate to the reptile, or the latter to the degree of irritability which obtains in the former, the functions of life would speedily become extinct. Various deviations from the usual proportion between the respiration and the irritability, however, occur, but there is an immediate tendency to restore that proportion; increased stimulus exhausts or lowers the degree of irritability, whilst diminished stimulus allows of its augmentation. The alternations between activity and sleep afford illustrations of these facts.

"Changes in anatomical form in the animal kingdom present other illustrations of the law of the inverse proportion of the respiration and irritability. The egg, the fœtus, the tadpole, the larva, &c. are respectively animals of lower respiration and of higher irritability than the same animals in their mature and perfect state. Changes in physiological condition also illustrate the same law. The conditions of lethargy, and of torpor, present examples of lower respiration and of higher irritability than the state of activity.

"It may be remarked that whilst changes in anatomical form are always from lower to higher conditions of existence, changes in the physiological condition are invariably from higher to lower." (P. 2.)

In order to verify the proposition that "*the quantity of the Respiration is inversely as the degree of the Irritability of the muscular fibre*," it became necessary to have some means of accurately measuring the consumption of oxygen in different animals, and under different circumstances. This was, indeed, a problem of no slight difficulty to solve, and the difficulties were, as our author observes, "such as to lead one of the first chemists of the present day to give up some similar inquiries in despair."

The apparatus devised by Dr. Hall does, however, effectually answer the end proposed: he has named it a "Pneumatometer." It imports us not minutely to describe its construction, which could scarcely be understood without reference to figures, and as the plans have been published in the Philosophical Transactions, and the apparatus can, of course, be obtained from any philosophical instrument maker, a republication of these would be a work of supererogation. Suffice it that we give the result of a series of experiments made with this instrument. But first

"*Of the Measure of the Irritability.* The problem to be next determined is that of the degree of irritability of the muscular fibre, and especially of the heart. This question is beset with scarcely fewer or less difficulties than that of the quantity of respiration, whilst it involves far greater errors and more discrepancy of opinion on the part of physiologists.

"Even Baron Cuvier has fallen into these errors. It will be shortly demonstrated that the degree of irritability is, in every instance, inversely as the quantity of respiration. Yet M. Cuvier, in a remarkable paragraph, states the very contrary, and even speaks of that which is the exhauster, as the repairer, of the irritability; whilst, on the other hand, he makes statements which appear to me at variance with this very opinion. In the Anatomie Comparée (tome i. p. 49,) this celebrated writer observes, 'Les expériences modernes ont montré qu'un des principaux usages de la respiration est de ranimer la force musculaire, en rendant à la fibre son irritabilité epuisée." See also tome iv. p. 301. Similar observations are made in M. Cuvier's more recent work, the Règne Animal: 'C'est de la respiration que les fibres musculaires tirent l'énergie de leur irritabilité;" tome i. p. 57, 2me edit. 'C'est la respiration qui donne au sang sa chaleur, et à la fibre la susceptibilitê pour l'irritation nerveuse;" tome ii. p. 1. On the other hand, speaking of the mollusca, (tome iii. p. 3,) M. Cuvier observes of those animals of low respiration, "L'irritabilité est extrême dans la plupart." The same term is, in fact, used in two distinct senses in these paragraphs.

"No further proof can be necessary of the extreme vagueness and incorrectness of the prevailing notions and expressions of physiologists in regard to this subject. All this will appear still more extraordinary when the law that the quantity of respiration and the degree of the irritability are, in fact, inverse throughout all the series, stages, and states of animated being, is clearly established.

"It is well known that the irritability of the heart and of the muscular fibre in general is greater in the mammalia than in birds, and in reptiles and the amphibia than in the mammalia, whether we judge of it by the force and duration of the beat of the heart, exposed to the stimulus of the atmospheric air, or by the contractions of the other parts of the muscular system. Now this is precisely the order of the quantity of respiration in these animals, as ascertained by the pneumatometer, inverted. It is essential, in accurately determining the question of the irritability of the muscular fibre, to compare animals of the same class inter se; birds and the mammalia, reptiles and the amphibia, fishes, the mollusca, &c. must be compared with each other, both generically and specifically. It is especially necessary to compare the warm-blooded, the cold-blooded, the air-breathers, and the water-breathers, in this manner. However the different classes may differ from each other, there are differences in some of the species of the same class, and especially that of fishes, scarcely less remarkable.

"Great difference in the duration of the beat of the heart are observed in the fœtal, early, and adult, states of the higher animals; this duration being greatest in the first, and least in the last of these conditions. The order of the quantity of respiration is inverse.

"The law of the irritability being inversely as the respiration, obtains even in the two sides of the heart itself in the higher classes of animals. The beat of the heart, removed from the body, does not cease at the same time in the walls of all its cavities, or of its two sides: but, as Harvey observes, 'primus desinit pulsare sinister ventriculus; deinde ejus auricula; demum dexter ventriculus; ultimo (quod etiam notavit Galenus) reliquis omnibus cessantibus et mortuis, pulsat usque dextra auricula.'

"Even in this case the irritability is greatest in the part in which the respiration is least." (P. 7.)

After detailing an experiment performed to shew the relative degree of irritability of the two ventricles of the heart, and by which it is proved that the irritability of the right ventricle is superior to that of the left, our author continues,

"It is quite obvious that the heart will bear a suspended respiration better, the more nearly its irritability approaches to that which may be designated veno-contractile. The power of bearing a suspended respiration thus becomes a measure of the irritability. It is expressed, numerically indeed, by the length of time during which the animal can support a suspended respiration; a conclusion of the highest degree of importance in the present inquiry.

"Birds die almost instantly on being submerged in water; the mammalia survive about three minutes, the reptiles and the batrachia a much greater length of time.

"The unborn fœtus, the young animal born with the foramen ovale open, the reptile, the mollusca, having all a state of the heart approaching to the veno-contractile, bear a long-continued suspension of the respiration, compared with the mature animal of the higher classes.

"But the most remarkable fact deducible from this reasoning is the following: if such a case existed as that of the left side of the heart being nearly or absolutely veno-contractile, such an animal would bear the indefinite suspension of respiration; such an animal would not drown though immersed in water. Now there is precisely such a case: it is that of the hybernating animal. It will be shewn in the subsequent paper, that, in the state of perfect hybernation, the respiration is nearly suspended; the blood must, therefore, be venous. Yet the heart continues to contract, although with a reptile slowness: the left ventricle is, therefore, veno-contractile, and, in this sense, in fact sub-reptile. The case forms a sole exception to the law pointed out by Harvey, that the left ventricle ceases to contract sooner than the right. If, in the hybernating animal, the left ventricle does cease to beat sooner than the right, it is only in so slight a degree as to be referred to the greater thickness of its parietes, and the slight degree in which respiration still remains. It is obvious that the foregoing statement must be taken with its due limitations. Venous blood is unfit for the other animal purposes, even though it should stimulate the heart to contraction." (P. 10.)

The following acute physiological criticism is particularly worthy of attention.

"There are two other properties of animals which depend upon the varied forms of the inverse ratio which exists between the respiration and the irritability. The first is *activity*, the second, *tenacity of life*.

"The activity (which, I believe, M. Cuvier has confounded with the irritability,) is generally directly proportionate to the respiration, and intimately depends upon the condition of the nervous system resulting from the impression of a highly arterial blood upon its masses, and not upon the degree of irritability of the muscular fibre. It is the pure effect of high stimulus.

"To shew that M. Cuvier has blended the idea of the irritability of the muscular fibre with that of the activity of the animal, it is only necessary to recur to the passages already quoted from that author, and to adduce the observations with which they are connected. 'On vient de voir à quel point les animaux vertébrés se ressemblent entre eux; ils offrent cependant quatre grandes subdivisions ou classes, caractérisées par l'espèce ou la force de leurs mouvements, qui dépendent elles-mêmes de la quantité de leur respiration, attendu que c'est de la respiration que les fibres musculaires tirent l'énergie de leur irritabilité.'* 'Comme c'est la respiration qui donne au sang sa chaleur, et à la fibre la susceptibilité pour l'irritation nerveuse, les reptiles ont le sang froid, et les forces musculaires moindres en totalité que les quadrupèdes, et à plus forte raison que les oiseaux; aussi n'exercent-ils guère que les mouvements du ramper et du nager; et, quoique plusieurs sautent et courent fort vite en certains moments, leurs habitudes sont généralement paresseuses, leur digestion excessivement lente,

* Le Règne Animal, tome i. p. 56, 57, 2me edit.

leurs sensations obtuses, et dans les pays froids ou tempérés, ils passent presque tous l'hiver en léthargie.'*

"It is extraordinary that M. Cuvier should have associated the elevated temperature of the blood with a high irritability of the muscular fibre, when they are uniformly separated in nature, and are, indeed, absolutely incompatible in themselves. The muscular fibre of the frog is so irritable, that it would instantly pass into a state of rigid and permanent contraction, if bathed with a fluid of the temperature of the blood of birds.†

"The same confusion of ideas on the subject of the activity of the animal and the irritability of the muscular fibre prevails, I believe, amongst our own physiologists: at least, in conversation with two, who may rank among the first, I found that they had uniformly considered the respiration and the irritability to be directly, instead of inversely, proportionate to each other.

"That singular and interesting property of the lower orders of animals termed tenacity of life is, on the other hand, distinctly associated with a high degree of irritability of the muscular fibre. This property may be defined as consisting of the power of sustaining the privation of respiration, the privation of food, various mutilations, divisions, &c. It is greater as we descend in the zoological scale. As activity depends upon the presence and condition of the spino-cerebral masses acted upon by arterial blood, tenacity of life depends upon the diminution or absence of these masses and of this highly arterialized blood, being greatest of all in those animals which approach a mere muscular structure. Almost the sole vital property then remaining is the irritability; and this property does not immediately suffer from division.

"It is possible to reduce some of the reptile tribes to a state approaching that of animals still lower in the scale, by removing, by very slow degrees, successive portions of the nervous masses. This is most readily done in animals in which the respiration is already low and the irritability high, as in the fœtus, in the very young animal, in the reptile, &c., as in the experiments of Legallois,‡ M. Serres,§ myself,|| &c.

"There is, even in animals most tenacious of life, one kind of mutilation, one kind of injury, not well borne. As the blood is in its lowest condition of stimulus, it cannot be withdrawn with impunity; frogs even soon perish if their blood be allowed to flow. As the irritability, on the other hand, is high, certain stimuli, as galvanism, slightly elevated temperatures, &c., are speedily fatal. The batrachia are promptly destroyed by immersion in water of a temperature of 108° of Fahr., and some fish and crustacea perish in great numbers under the influence of a thunder-storm. It is a

* Le Règne Animal, tome ii. p. 1, 2, 2me edit.

† See an Essay on the Circulation, chap. vii. pp. 180, 181.

‡ Experiences sur le Principe de la Vie.

§ Anatomie Comparée du Cerveau, tome ii. p. 224.

|| Essay on the Circulation, chap. iii. § 1.

singular fact, that the fish alone whose food is found amongst animals of a high irritability, should possess an electrical organ for the destruction of its prey." (P. 11.)

In our next number we shall give an abstract from the interesting memoir, by Dr. Hall, "on Hybernation," which has also been recently published in the Philosophical Transactions.

